# Should I Stay or Should I Go? A Habitat-Dependent Dispersal Kernel Improves Prediction of Movement

**DOI:** 10.1371/journal.pone.0021115

**Published:** 2011-07-12

**Authors:** Fabrice Vinatier, Françoise Lescourret, Pierre-François Duyck, Olivier Martin, Rachid Senoussi, Philippe Tixier

**Affiliations:** 1 CIRAD, UPR26, Le Lamentin, Martinique; 2 INRA, UR 1115, Avignon, France; 3 INRA, UR 546, Avignon, France; Texas A&M University, United States of America

## Abstract

The analysis of animal movement within different landscapes may increase our understanding of how landscape features affect the perceptual range of animals. Perceptual range is linked to movement probability of an animal via a dispersal kernel, the latter being generally considered as spatially invariant but could be spatially affected. We hypothesize that spatial plasticity of an animal's dispersal kernel could greatly modify its distribution in time and space. After radio tracking the movements of walking insects (*Cosmopolites sordidus*) in banana plantations, we considered the movements of individuals as states of a Markov chain whose transition probabilities depended on the habitat characteristics of current and target locations. Combining a likelihood procedure and pattern-oriented modelling, we tested the hypothesis that dispersal kernel depended on habitat features. Our results were consistent with the concept that animal dispersal kernel depends on habitat features. Recognizing the plasticity of animal movement probabilities will provide insight into landscape-level ecological processes.

## Introduction

Animals generally combine a wide variety of chemical, visual, and acoustic cues to assess the suitability of habitats for providing food [1], oviposition sites [2], or protection from predators [3]. The perceptual range of an animal, i.e., the spatial extent of the landscape for which information is available to drive decisions about movement, is a determinant of the dynamics and spatial distribution of animal populations [4]. An animal's perceptual range is directly linked to landscape connectivity, and analysis of perceptual range can help researchers understand how populations respond to habitat disturbance and fragmentation [5]. Perceptual range is a key parameter of the probability that animals successfully disperse in a landscape, and consequently of the existence and persistence of a fragmented population [6]. Perceptual abilities drive the foraging behaviour of predators with respect to a spatially and temporally varying distribution of prey [7] as well as the population dynamics of pests such as crickets [8]. Mechanisms of habitat selection by large mammals and birds are also quite related to their perceptual ranges [9]–[11].

Several spatio-temporal discrete models define the concept of perceptual range through the description of an individual's habitat preference and animal movement analysis [12],[13]. In these models, the perceptual range of an individual represents an “information window” onto the surrounding landscape, where all potential habitats are given an availability coefficient either uniformly defined [9] or non-uniformly defined with a “dispersal kernel”. The dispersal kernel generally accounts for the relative cost of a movement (displacement) from one location to another in terms of the distance between locations and their ecological features [13], [14]. The class of useful dispersal kernels is rich and may accommodate various shapes that can be fixed on the basis of some *a priori* knowledge [15]. Simple and interpretable kernels can be made very flexible by adjustment of parameters whose values govern important indices of the spatial distribution of individuals [16]. For example, “fat-tailed” distributions or kernels allow long-distance dispersal events and generally describe large-scale colonisation processes in accordance with a large perceptual range of individuals [17].

Although animal dispersal kernel is traditionally taken as species-invariant [4], observational evidence indicates that it can be variable [18]. Some of the factors that can cause variation in the dispersal kernel among individuals of a species or population are intrinsic characteristics such as sex, age, social status, and energy reserves; environmental conditions such as climate, season, and habitat quality; and ecological characteristics such as levels of competition, predation, and parasitism [7], [19]. Other extrinsic environmental stimuli may also alter an animal's dispersal kernel [20]. Zollner and Lima [21] reported that the movement probabilities of white-footed mice significantly changes depending on whether they are released in bare fields or crop fields. In spite of its theoretical and practical significance [22], [23], the plasticity of animal movement probabilities in landscapes remains an unexplored research area [4]. It clearly deserves more theoretical and empirical investigation because appropriate estimation of dispersal kernel plasticity may lead to a better assessment of the functional connectivity of landscapes [24].

To assess whether and to what extent animal movement probability can be affected by spatial heterogeneity of habitats, we considered a data set of the locations of the insect *Cosmopolites sordidus* (coleoptera) within heterogeneous environments [25]. For that purpose, we used recent advances in radio-tracking techniques [26] to monitor the fine-scale movements of over 1000 individuals in five banana plots.

In this study, we assumed that the movement probability is defined by a negative-exponential kernel with a single parameter *β* that may account for the influence of the habitat features of the current animal location before a displacement. We then proposed a discrete space–time stochastic model of animal movement as a Markov chain in which the movement between arrival and departure locations depends on their geographic distance and possibly on their respective habitat characteristics. For our analysis, we considered the particular hypothesis H_0_ of a habitat-independent kernel (*β* independent of the habitat type of the departure cell) versus the general hypothesis H_1_ of a habitat-dependent kernel (*β* dependent on the habitat type of the departure cell). Using a radio-tracking data set of *C. sordidus* movements, we first tested the sub-model H_0_ against H_1_ with the likelihood ratio test. To reinforce our results, we then applied the pattern-oriented modelling (POM) approach [27] to compare the two hypotheses with spatially explicit simulations of the respective underlying individual-based models. POM is a general validation procedure that focuses on the analysis of pertinent variables, e.g., an animal's use of space. POM is based on the emerging recognition that population-level patterns may result from individual behaviours [28]. The POM procedure can thus help unravel the effects of different implicit or explicit assumptions underlying ecological models. In our study, POM is based on the simulation of the alternative models calibrated with their respective maximum likelihood estimates of parameters. Discrimination of the two models relies on testing their ability to reproduce the patterns observed in the studied plots with respect to two pertinent ecological variables [27], which are the proportion of non-moving individuals and the distribution of displacement lengths.

## Materials and Methods

### Materials: species, plots, and radio tracking

The banana weevil, *Cosmopolites sordidus*, is a walking insect with cryptic and nocturnal activities. It lives in all countries where its only host plant, the banana, grows [29]. Adults prefer moist environments and feed on banana plants or their residues. Females lay eggs at the base of the host plant, and larvae grow inside the corm. The movements of *C. sordidus* are not known to be socially organized or to be dependent on gender [25].

Daily radio-tracking data were collected for approximately 600 males and 600 females of wild *C. sordidus* that were caught with pseudostem traps from one banana field adjacent to the study site (Table S1). Insects caught were sexed and kept in laboratory approximately one week before release. They were tagged two hours before release using passive radio-tracking tags, released in five banana plots and followed for at least 10 days (for more details on the radio-tracking method, see [25]). Field studies were conducted according to the “Pôle de Recherche Agro-environnemental de la Martinique” permission. Each plot was depicted as a regular lattice of 800 to 2400 cells of 1-m^2^. This cell size was chosen because it was small enough to characterize resource variability [30] and large enough to match radio-tracking accuracy [25]. Locations of individuals were rounded to one-meter grain and pinpointed at cell centres. Regular space–time agricultural practices on banana plots result in the occurrence of a structured mosaic of habitats (Figure S1). We distinguished four mutually exclusive types of habitat: (*P*) host plant, (*C*) crop residue, (*B*) bare soil, and (*D*) ditch. Types *P* and *C* are recognized as more suitable habitats for *C. sordidus* than *B* and *D.* Plots 3–5 contained a high proportion of suitable habitats while plots 1–2 contained a high proportion of unsuitable habitats (Figure S1).

### Methods: Discrete space–time stochastic modelling

To describe beetle movement in a plot, we chose a stochastic and discrete space–time formalism following an individual-based model developed earlier for this pest [31]. The spatial environment was represented by a lattice of *n* cells. Each cell *i* (*i = 1,…,n*) was characterised by its centre coordinates *c_i_ = (x_i_, y_i_)* and its habitat type *h_i_ (h_i_ = P, C, B, D)*. Individual movements were considered as a Markovian random walk on the lattice centres. More specifically, we assumed that individuals moved independently from each other and that individuals had no memory of their previous displacements. We also assumed that the daily decision to remain in a cell or move from a cell was independent of time but depended on the habitat quality of this cell and on the attractiveness and closeness of other cells. With this time-homogeneous Markovian hypothesis, we considered *C. sordidus* walks as a first-order Markov chain whose states corresponded to cell centres and whose transition probabilities were defined with a dispersal kernel 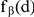. An exponential form for the dispersal kernel, 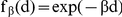, was selected because of its simplicity and ease of interpretation. We allowed the shape coefficient β, however, to depend on the habitat type of departure cells. We expressed the daily probability of moving from the current cell *c_i_* to an arrival cell *c_j_* as:
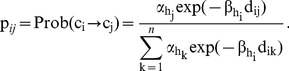
(1)where *d_ij_ = d(c_i,_, c_j_)* is the Euclidean distance between centres of cells i and j. The parameters 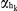, which are non-negative and satisfy equality 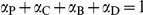, can be interpreted as the relative attractiveness of habitat type *h_k_* of cell k. The parameters 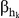 are non-negative and can be linked to the mean sojourn time in habitat type *h_k_*, as explained below.

The Markovian hypothesis implies that the sojourn time τ_i_ in a cell *i* has a geometric distribution with parameter *p_ii_*. The mean sojourn time is a function of parameters α and β and of all distances *d_ik_* between cell *i* and other cells:
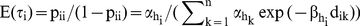
(2)It implies that the probability of staying in a given cell (i.e. *p_ii_*) is different from one, especially for cells containing a good habitat, such as banana plants or crop residues. Ecologically, it means that animals located in good habitats move because they need to change place for egg-laying and/or mating during the study period.

To understand the intrinsic role of parameter β, we assumed that α is constant and that the number of cells is large enough so that the following approximation can be used:
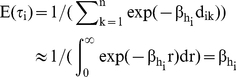
(3)


### Likelihood formula

Because individuals were independent and individual movements were Markovian, the data likelihood within a single plot *p* consisted of the product of the daily movement probabilities according to equation [1] over all individuals released in this plot *(m = 1,…,M)* and over all their daily moves *c(m,t)→c(m,t+1)*; *t = 0,…,T_m_−1* where *T_m_* was the observation period of individual *m*:
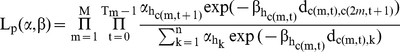
(4)and *c(m,t)* denoted the location (cell centre) of individual *m* at time *t*, *h_c(m,t)_* denoted its habitat type, and *n* denoted the number of cells of the plot. Actually, given the hypothesis that model parameters were independent of the five plots *(p = 1 to 5)*, the final likelihood is:
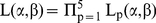
(5)


### Maximum likelihood estimation and hypothesis testing

First, we allowed the parameters α_h_ and β_h_ to take distinct values for the four distinct habitats in what we called the general model (denoted M_G_^4^) and estimated the parameters by a maximum log-likelihood procedure of L(α, β). For that purpose, we used Nelder's Mead algorithm [32], which accounted for the parameter positiveness and the α's constraint (α_P_+α_C_+α_B_+α_D_ = *1*). We also considered different sub-models (or hypotheses) in which some of the α's (respectively β) parameters were set equal, e.g., α_P_ = α_C_ = α_P+C_ (respectively β_P_ = β_C_ = β_P+C_), which eventually amounted to the grouping of habitat *P* and *C* into a single type. We consequently denoted such sub-models as, e.g., M_G_^3,(P+C)^ and used the same procedure and algorithm for parameter estimation.

To test data fit of models M_G_ (with k_G_ parameters) and M_0_ (with k_0_ parameters, sub-model [nested model] of M_G_), we used the classical likelihood ratio statistic 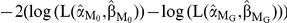, which was expected to follow under M_0_ a χ^2^ distribution with df = k_G_−k_0_. Within different habitat regrouping contexts, we might have tested a large number of nested hypotheses. For simplicity, we consider only the reasonable alternative hypotheses of habitat-independent moves (H_0_: β_h_ is independent of h, α_h_ are distinct) versus habitat-dependent moves (H_1_: β_h_ are distinct, α_h_ are distinct).

### Pattern-oriented modelling

POM can be considered as a validation procedure for a specified model and is used to reproduce important patterns or statistical characteristics of a specific process [27]. POM consisted of developing and then simulating a spatially and temporally explicit individual-based model and assuming many mechanistic hypotheses. Some model outputs were then statistically compared to those of an observed data set. Discrepant results would indicate the irrelevance or omission of important working hypotheses.

In this study, we used the maximum likelihood estimates for model M_G_^4^ (respectively M_0_^4^, see *Maximum likelihood estimation and hypothesis testing*) of a habitat-dependent (respectively independent) dispersal kernel to simulate 100 runs of the walk of the original *C. sordidus* population within the five plots. At each run and for each plot, all individuals of the population were spatially distributed according to their observed released position. The simulations covered 10 days. Data simulated from the two models were then compared to radio-tracking observations, with focus on two pertinent ecological variables of space use by animals. The first variable refers to the proportion of individuals remaining in their release cell throughout the study period. This variable might characterise the tendency of *C. sordidus* to be sedentary unless motivated to move by significant differences in environment suitability. The second variable describes the distribution of dispersal distances that characterise *C. sordidus* mobility and that depend on both soil roughness and habitat diversity. We restricted our analysis to two patterns able to discriminate between the two models. Other patterns such as direction of movements depend mainly on relative attractiveness of habitat and not on dispersal kernel, and mean squared displacement of movement is highly correlated to the distribution of dispersal distances.

Each simulated variable was represented by the mean of 100 runs, and the simulated and observed means were compared with the classical χ2 test statistic for proportions and with the Kolmogorov-Smirnov test for distance distributions [33].

## Results

Regardless of the number of distinct habitats considered, the target habitat-preference estimates (the α's attractiveness coefficients) remained similar and their relative ranking remained very stable for the habitat-independent M_0_ and habitat-dependent models M_G_. More specifically, host plant (P) and crop residue (C) habitats were always highly and equally preferred over bare soil (B) and ditch (D) habitats (Table 1). When the four habitat types were dissociated, the log-likelihood of the habitat-dependent model M_G_^4^ was significantly greater than that of the habitat-independent model M_0_^4^ (Table 1, χ^2^_3_ = 597, *p*<0.001). When habitat types were pooled, the log-likelihood naturally decreased with parameter dimension for both models; note that Table 1 gives the opposite log-likelihood value. Also note, however, that the log-likelihood remained similar for the habitat-dependent models M_G_^4^ and M_G_^3^ (resp. the habitat independent models M_0_^4^ and M_0_^3^) when host plant (P) and crop residue (C) habitats were pooled (χ^2^_2_ = 1, *p* = 0.61) (resp. χ^2^_1_, *p* = 0.5). In all other cases, the embedded sub-models were significantly rejected (χ^2^_1 to 3_ tests *p*<0.001). All habitat-dependent models M_G_^k^ (k = 3 (P+C), 3 (D+B), 2) performed significantly better than the habitat-independent models M_0_^k^ (M_0/G_^3,(P+C)^: χ^2^_2_ = 596, M_0/G_^3,(D+B)^: χ^2^_2_ = 571, M_0/G_^2^: χ^2^_1_ = 569, *p*<0.001 in all cases).

**Table 1:** Modified log-likelihood [−2log(L)] and parameter estimates for the different models.

	Dispersal kernel parameters *β_h_*					Preference parameters *α_h_*				df	−2.log(L)
4 habitats											
		*β_P_*	*β_C_*	*β_B_*	*β_D_*	*α_P_*	*α_C_*	*α_B_*	*α_D_*		
M_0_^4^	1.62					0.54	0.43	0.018	0.008	4	12991
M_G_^4^		*2.01*	*2.11*	*1.14*	*0.71*	*0.54*	*0.40*	*0.036*	*0.014*	*7*	***12394***
3 habitats (grouping Host plant+Crop residue)											
		*β_P+C_*	*β_B_*	*β_D_*		*α_P+C_*	*α_B_*	*α_D_*			
M_0_^3,(P+C)^	1.63					0.95	0.01	0.04		3	12991
M_G_^3,(P+C)^		*2.04*	*1.09*	*0.74*		*0.91*	*0.02*	*0.07*		*5*	***12395***
3 habitats (grouping Ditch+Bare soil)											
		*β_P_*	*β_C_*	*β_B+D_*		*α_P_*	*α_C_*	*α_B+D_*			
M_0_^3,(B+D)^	1.62					0.55	0.43	0.02		3	12993
M_G_^3,(B+D)^		*1.97*	*2.14*	*1.08*		*0.56*	*0.41*	*0.03*		*5*	***12422***
2 habitats (grouping Host plant+Crop residue and Ditch+Bare soil)											
		*β_P+C_*	*β_B+D_*			*α_P+C_*	*α_B+D_*				
M_0_^2^	1.63					0.97	0.03			2	13014
M_G_^2^		*2.04*	*1.08*			*0.94*	*0.06*			*3*	***12445***
1 single habitat (grouping Host plant+Crop residue+Ditch+Bare soil)											
		*β_P+C+B+D_*				*α_P+C+B+D_*					
M_G_^1^ = M_G_^2^		1.89				0.25				1	14769

Parameter subscripts: P, host plant; C, crop residue (litter-covered soil); D, ditch; B, bare soil. P+C means that host plant and crop residue habitats are pooled in a single category.

Habitat-dependent (resp. habitat-independent) models with k types of habitat are denoted M_G_^k^ (resp. M_0_^k^).

**Figure 1:**
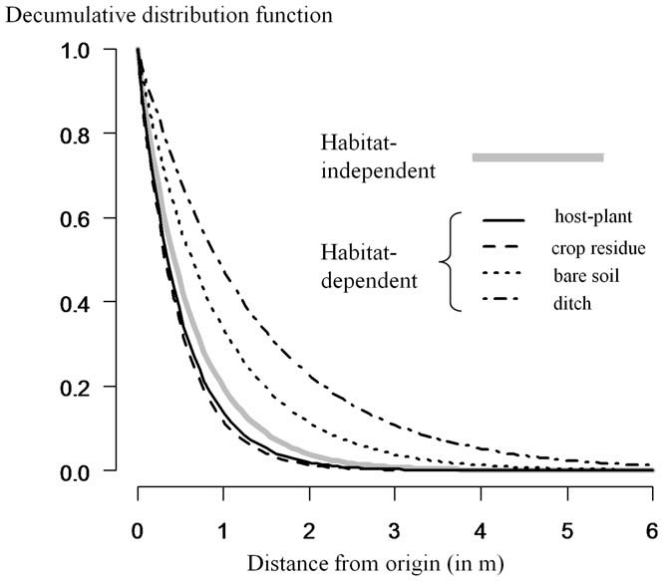
The “décumulative” distribution function as a function of length of animal displacement (d), i.e., *f(d) = exp(−β.d)*. Maximum likelihood estimates of exponential kernels are drawn for the habitat-independent (grey line) and the habitat-dependent (black lines) models. The slopes of the black curves depend on the estimated value of parameter *β*_h_ for the habitat type h of the individual's location before movement.

As indicated earlier, the *β* parameter values define the shape of the dispersal kernel assigned for each habitat feature and can be interpreted as the mean sojourn time in the current location when the attractiveness parameters α's of habitats are equal: the higher the *β_h_* value for the current habitat *h*, the higher the tendency for the individual to remain in cells of habitat type *h*. Maximum likelihood estimators of *β_h_* were high for the host plant (P) or the crop residue (C), intermediate for the bare soil (B), and low for the ditch (D) (Table 1). This means that the probability of movement was high if the current habitat was ditch (D), was intermediate if the current habitat was bare soil (D), and was low if the current habitat was crop residue (C) or host plant (P) (Fig. 1). Note the almost constant value of parameter *β* (≈1.62) for all habitat-independent models M_0_^k^ regardless of how habitat types were grouped (Table 1).

**Figure 2:**
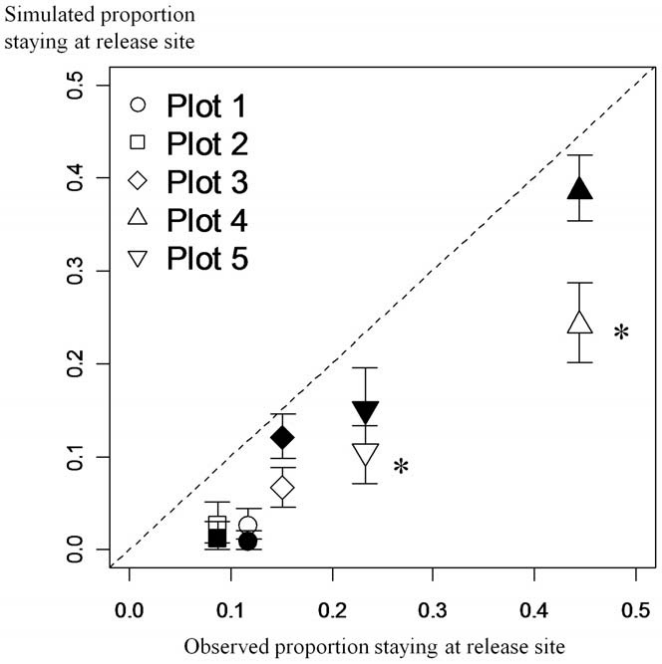
Proportion of individuals staying at their release site in the five banana plots (observed versus simulated by POM). Means for each of the five banana plots and 95% quantile interval (vertical bars) were calculated for the habitat-independent (white) and the habitat-dependent (black) models with the respective maximum likelihood estimates based on 100 runs. The dotted line corresponds to ideal fit between observations and simulations. For each plot, an asterisk indicates a significant difference between the simulated and observed mean (χ^2^ test, df = 1, P<0.01).

**Figure 3:**
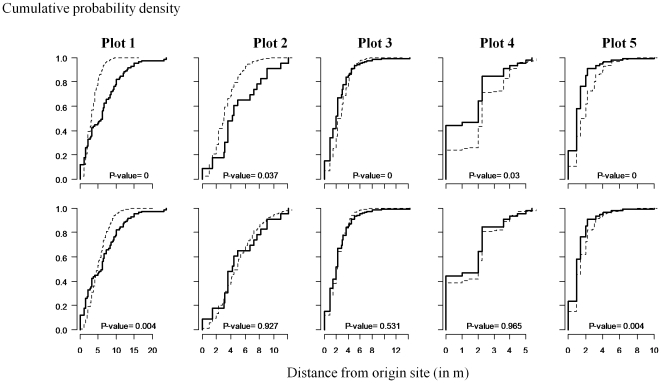
Dispersal distances in the five bananas plots (observed versus simulated by POM). Simulations were driven for habitat-dependent and habitat-independent models with the respective maximum likelihood estimates. Cumulative distribution plot of simulated distances (dashed line) versus observed distances (bold line) from (a) the habitat-independent model and (b) the habitat-dependent model. P-values correspond to the results of the Kolmogorov-Smirnov test of equal distributions for observed and simulated dispersal distances (based on >100 simulations).

Concerning the POM procedure, the habitat-independent model M_0_^4^ significantly underestimated the proportion of individuals remaining in their release cells in plots 4 and 5 (Fig. 2). The habitat-independent model M_0_^4^ overestimated the dispersal distances in all plots except plot 1 and 2, in which it underestimated the distance distribution (Fig. 3). In contrast, the habitat-dependent model M_G_^4^ accurately reproduced the two characteristics of space use. The Kolmogorov-Smirnov test, however, rejected the hypothesis of equal distribution for observed and simulated dispersal distances in plots 1 and 5 despite the closeness of the two distributions (Fig. 3).

## Discussion

In this study of movement probabilities of the walking insect *C. sordidus*, we developed a stochastic Markov model to explore the effect of both target and departure habitats on an animal's decision to move or not to move. The ranking of “immigration attractiveness coefficients of habitats” (the *α*_h_ parameters) was consistent with the *a priori* ordering of habitat quality for *C. sordidus*: the host plant is the most attractive for feeding and egg laying, and the litter-covered soil (crop residue) is the most attractive for protection against predators and feeding. Bare soil and ditch are less attractive because they are drier, provide no food, and offer no physical protection against predators. The ranking of the “habitat sedentariness coefficients” (the *β*_h_ parameters), which describe the cost of departure from a habitat, was similar to that of the *α*_h_ coefficients. This concordance simply indicated that preferred habitats were those with high values for the coefficients α and β, i.e., those which *C. sordidus* remained within or moved to.

Rhodes et al. [13] emphasized that animal movement probabilities could be usefully described as a function of habitat. Our results clearly support this view and showed that incorporating habitat dependency in dispersal kernels of spatially explicit models greatly improves our understanding of animal movements. Furthermore, the complementary POM approach showed that habitat-independent models failed to describe two pertinent statistical characteristics of animal space use. Introducing a habitat-dependent dispersal kernel was found to be useful and relevant because it enabled a reasonable statistical replication of the spatial and temporal behaviour of animals in habitats of both low and high suitability. Our study, therefore, provides elements to respond to the call by Olden et al. [4] for the development of spatially explicit models of animal movements that integrate the concept of context-dependent perceptual ranges.

Lima and Zollner [5] pointed out that perceptual range strongly depends on species and represents a key trait of mortality risk of dispersing animals. The authors reported that animals with high perceptual range are subjected to a higher risk of mortality because they spend more time searching suitable habitat. On the one hand, our results confirmed that individuals located in unsuitable habitats (bare soil or ditch) and experiencing a high risk of predation consequently “increase” their movement probabilities to perceive distant protective habitats such as host plant or litter-covered soil. On the other hand, the study also showed that individuals located in suitable habitats with a low mortality risk might “reduce” their movement probabilities and stay longer on these favourable sites. Our analysis emphasized that individuals adapt their displacements depending on their current locations. This is in accordance with Huffaker and Gutierrez [3], who argued that many insects adapt their movement traits to optimize their presence in favourable areas. However, using a simulation model, Zollner and Lima [34] found a minor role of landscape configuration on behavioural tradeoffs between perceptual range and predation risk. Authors concluded that the shape of the relationships between perceptual range and predation risk was the main factor affecting dispersal success of animals.

The use of simple mechanistic models like the one presented here might clarify complex processes such as the plasticity of movement. The dispersal kernel of *C. sordidus* appeared more extended in bare soil than in banana plants. Zollner and Lima [21] found the same result with white-footed mice, and they hypothesized that these forest mice might locate suitable habitats by mainly using vision: their perceptual range was large in bare fields, which lacked visual obstructions, but small in crop fields, which contained many visual obstructions. *Cosmopolites sordidus*, in contrast, would perceive its environment through semiochemical stimuli [29], and we might interpret that the alteration of its dispersal kernel on a banana plant habitat was due to a saturation of the environment by local attractive chemicals. Conversely, bare soils contained only low concentrations of local chemical attractants, and in such an environment *C. sordidus* might be more responsive to surrounding cues.

Our dispersal model relies on two substantial assumptions. The first assumption is that individuals have no social interaction and behave independently of each other. This assumption appears to be justified because the discrete choice model did not contain a social component but correctly described the distribution of observed displacement lengths. The second assumption is that daily moves of an individual are independent of each other. This is the Markovian property of our model and is generally called ‘first-order memory loss’. We might consider this assumption as reasonable because *C. sordidus* individuals rest during the day and move at night. In other contexts and for other species, animal walks are correlated, i.e., moves are sequentially related to each other [35]. For such cases, more complex (second- or third-order Markovian models) could be built to describe two or three consecutive displacements. Another refinement would be to model not only the length but also the direction (angle) of displacements when it is pertinent, e.g., in response to wind direction, sun light, altitude, etc.

Our results illustrate that likelihood- and POM-based approaches are complementary and can be used to increase the understanding of ecological processes. In most contexts, however, these approaches have different uses. Likelihood procedures are more suitable for comparing empirical and parsimonious statistical sub-models. POM procedures, in contrast, are more suitable for comparing mechanistic models based on their ability to simulate observed patterns. Models using POM procedures may contain numerous deterministic and stochastic mechanisms that cannot be handled by a statistical formulation. This perhaps explains why these complementary methods are rarely used by the same community of scientists [36]. The POM approach might be correctly considered as a way to validate a model by simultaneously addressing many characteristics of a complex process. The dispersal model developed in this study is simple enough to enable a tractable statistical formalism and rich enough to allow the emergence of properties of space use at the population level. This illustrates the value of a trade-off between simplicity and complexity in ecological studies.

## Supporting Information

Table S1 **Characteristics of the radio-tracking data sets.** Adults of *C. sordidus* were trapped in the field near their release site. They were sexed and marked using passive RFID (radio-frequency identification) tags. A preliminary study in controlled conditions indicated that tags did not affect adult movement. After the adults were released in the plots, their positions were checked daily with a recapture rate ranging from 50 to 80% and a precision of the position of 30 cm. *C. sordidus* movement is highly variable between individuals and between days, and ranges from 0 to 900 cm in one night. We extracted only relocations separated by 1 day and during the first week for analysis. This led to 3388 pairs of radio-tracking locations. Locations defined in decimetres were rounded to the proximate meter in order to have each position located in the centre of a given cell of the raster grid.
(DOC)

Figure S1 **Plot-raster of the five habitats used for the** ***Cosmopolites sordidus*** **movement study.** Each cell is a 1-m^2^ square. Plots 1 and 2 are composed mainly of bare soil. The proportions of host plant and crop residues are larger in Plots 3–5 than in Plots 1–2. Host plants are planted in staggered rows in Plots 3 and 4, with a cover of crop residues in Plot 3. In Plot 5, host plants are planted in 10 irregular double-rows, with an irregular cover of crop residues between host plants in each double-row. Banana plantations are composed of a matrix of heterogeneous habitats likely to influence *Cosmopolites sordidus* movements. Banana plants are considered as semi-perennial because plants are successively replaced by suckers emerging at irregular intervals from the lateral shoots of the mother plant, leading to almost 10 cropping cycles before destruction of the field. Each host plant is a mat consisting of a mother plant, a shoot, and an old plant. At the end of the first cropping cycle, banana leaves and other crop residues are cut and form a permanent litter cover on the soil. Ditches about 80 cm deep are formed to increase drainage. To characterize the environment of each plot, we considered that each plot consisted of a raster grid of 1-m×1-m cells with the value of each cell representing the most common habitat in the cell.
(DOC)
