# Supplementary material for: Should I Stay or Should I Go? A Habitat-Dependent Dispersal Kernel Improves Prediction of Movement
Source: PLoS One. 2011 Jul 12;6(7):e21115. doi: 10.1371/journal.pone.0021115 (PMC3134457; doi:10.1371/journal.pone.0021115)
Supplement: Table S1 — Characteristics of the radio-tracking data sets. Adults of C. sordidus were trapped in the field near their release site. They were sexed and marked using passive RFID (radio-frequency identification) tags. A preliminary study in controlled conditions indicated that tags did not affect adult movement. After the adults were released in the plots, their positions were checked daily with a recapture rate ranging from 50 to 80% and a precision of the position of 30 cm. C. sordidus movement is highly variable between individuals and between days, and ranges from 0 to 900 cm in one night. We extracted only relocations separated by 1 day and during the first week for analysis. This led to 3388 pairs of radio-tracking locations. Locations defined in decimetres were rounded to the proximate meter in order to have each position located in the centre of a given cell of the raster grid. (DOC) [file pone.0021115.s001.doc]

Table S1. Characteristics of the radio-tracking data sets. Adults of *C. sordidus* were trapped in the field near their release site. They were sexed and marked using passive RFID (radio-frequency identification) tags. A preliminary study in controlled conditions indicated that tags did not affect adult movement. After the adults were released in the plots, their positions were checked daily with a recapture rate ranging from 50 to 80% and a precision of the position of 30 cm. *C. sordidus* movement is highly variable between individuals and between days, and ranges from 0 to 900 cm in one night. We extracted only relocations separated by 1 day and during the first week for analysis. This led to 3388 pairs of radio-tracking locations. Locations defined in decimetres were rounded to the proximate meter in order to have each position located in the centre of a given cell of the raster grid.

Plot 1 Plot 2 Plot 3 Plot 4 Plot 5

Localisation

14°37'N, 60°58'W 14°37'N, 60°58'W 14°37'N, 60°58'W 14°37'N, 60°58'W 14°39'N, 60°58'W

Area

800 m^2^ 1600 m^2^ 1300 m^2^ 1300 m^2^ 2400 m^2^

Period of study

September 2009 December 2009 July 2009 May 2009 January 2009

Effective length of study

10 days 11 days 29 days 22 days 38 days

Number of released weevils

261 144 360 360 204

Number of consecutive recaptures

544 228 1071 572 973
