# Supplementary material for: Should I Stay or Should I Go? A Habitat-Dependent Dispersal Kernel Improves Prediction of Movement
Source: PLoS One. 2011 Jul 12;6(7):e21115. doi: 10.1371/journal.pone.0021115 (PMC3134457; doi:10.1371/journal.pone.0021115)
Supplement: Figure S1 — Plot-raster of the five habitats used for the Cosmopolites sordidus movement study. Each cell is a 1-m2 square. Plots 1 and 2 are composed mainly of bare soil. The proportions of host plant and crop residues are larger in Plots 3–5 than in Plots 1–2. Host plants are planted in staggered rows in Plots 3 and 4, with a cover of crop residues in Plot 3. In Plot 5, host plants are planted in 10 irregular double-rows, with an irregular cover of crop residues between host plants in each double-row. Banana plantations are composed of a matrix of heterogeneous habitats likely to influence Cosmopolites sordidus movements. Banana plants are considered as semi-perennial because plants are successively replaced by suckers emerging at irregular intervals from the lateral shoots of the mother plant, leading to almost 10 cropping cycles before destruction of the field. Each host plant is a mat consisting of a mother plant, a shoot, and an old plant. At the end of the first cropping cycle, banana leaves and other crop residues are cut and form a permanent litter cover on the soil. Ditches about 80 cm deep are formed to increase drainage. To characterize the environment of each plot, we considered that each plot consisted of a raster grid of 1-m×1-m cells with the value of each cell representing the most common habitat in the cell. (DOC) [file pone.0021115.s002.doc]

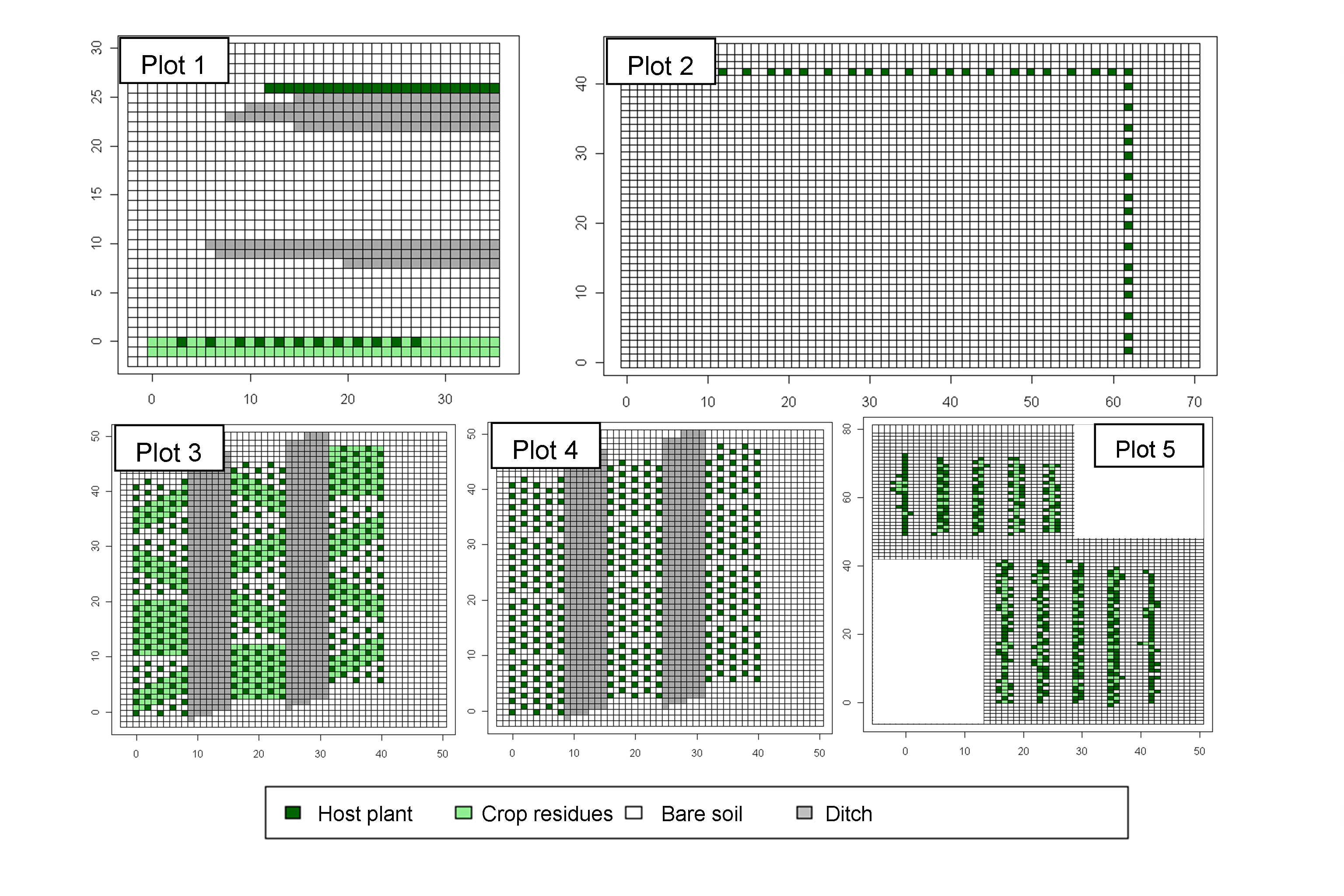


Figure S1. Plot-raster of the five habitats used for the *Cosmopolites sordidus* movement study. Each cell is a 1-m^2^ square. Plots 1 and 2 are composed mainly of bare soil. The proportions of host plant and crop residues are larger in Plots 3-5 than in Plots 1-2. Host plants are planted in staggered rows in Plots 3 and 4, with a cover of crop residues in Plot 3. In Plot 5, host plants are planted in 10 irregular double-rows, with an irregular cover of crop residues between host plants in each double-row. Banana plantations are composed of a matrix of heterogeneous habitats likely to influence *Cosmopolites sordidus* movements. Banana plants are considered as semi-perennial because plants are successively replaced by suckers emerging at irregular intervals from the lateral shoots of the mother plant, leading to almost 10 cropping cycles before destruction of the field. Each host plant is a mat consisting of a mother plant, a shoot, and an old plant. At the end of the first cropping cycle, banana leaves and other crop residues are cut and form a permanent litter cover on the soil. Ditches about 80 cm deep are formed to increase drainage. To characterize the environment of each plot, we considered that each plot consisted of a raster grid of 1-m x 1-m cells with the value of each cell representing the most common habitat in the cell.
